# 4-(Azolyl)-Benzamidines as a Novel Chemotype for ASIC1a Inhibitors

**DOI:** 10.3390/ijms25073584

**Published:** 2024-03-22

**Authors:** Maksym Platonov, Oleksandr Maximyuk, Alexey Rayevsky, Vasyl Hurmach, Olena Iegorova, Vasyl Naumchyk, Elijah Bulgakov, Andrii Cherninskyi, Danil Ozheredov, Serhiy V. Ryabukhin, Oleg Krishtal, Dmytro M. Volochnyuk

**Affiliations:** 1 Institute of Molecular Biology and Genetics, National Academy of Sciences of Ukraine, Zabolotnogo Str., 150, 03143 Kyiv, Ukraine; platon1971@gmail.com (M.P.); gyrmach@gmail.com (V.H.); 2 Enamine Ltd., 78 Winston Churchill Str., 02660 Kyiv, Ukraine; vasyl.naumchyk@gmail.com (V.N.); elihbul@gmail.com (E.B.); d.volochnyuk@gmail.com (D.M.V.); 3 Bogomoletz Institute of Physiology, National Academy of Sciences of Ukraine, 4 Bogomoletz Str., 01024 Kyiv, Ukraine; am@biph.kiev.ua (O.M.); egorova@biph.kiev.ua (O.I.); andrii.cherninskyi@biph.kiev.ua (A.C.); krishtal@biph.kiev.ua (O.K.); 4 Institute of Food Biotechnology and Genomics, National Academy of Sciences of Ukraine, Osypovskoho Str., 2A, 04123 Kyiv, Ukraine; ozheredovdanil@gmail.com; 5 Institute of High Technologies, Taras Shevchenko National University of Kyiv, Volodymyrska Street 60, 01601 Kyiv, Ukraine; 6 Institute of Organic Chemistry, National Academy of Sciences of Ukraine, 5 Academik Kukhar Str., 02660 Kyiv, Ukraine

**Keywords:** acid-sensitive ion channel, virtual screening, drug discovery, screening assay, automated patch clamp

## Abstract

Acid-sensing ion channels (ASICs) play a key role in the perception and response to extracellular acidification changes. These proton-gated cation channels are critical for neuronal functions, like learning and memory, fear, mechanosensation and internal adjustments like synaptic plasticity. Moreover, they play a key role in neuronal degeneration, ischemic neuronal injury, seizure termination, pain-sensing, etc. Functional ASICs are homo or heterotrimers formed with (ASIC1–ASIC3) homologous subunits. ASIC1a, a major ASIC isoform in the central nervous system (CNS), possesses an acidic pocket in the extracellular region, which is a key regulator of channel gating. Growing data suggest that ASIC1a channels are a potential therapeutic target for treating a variety of neurological disorders, including stroke, epilepsy and pain. Many studies were aimed at identifying allosteric modulators of ASIC channels. However, the regulation of ASICs remains poorly understood. Using all available crystal structures, which correspond to different functional states of ASIC1, and a molecular dynamics simulation (MD) protocol, we analyzed the process of channel inactivation. Then we applied a molecular docking procedure to predict the protein conformation suitable for the amiloride binding. To confirm the effect of its sole active blocker against the ASIC1 state transition route we studied the complex with another MD simulation run. Further experiments evaluated various compounds in the Enamine library that emerge with a detectable ASIC inhibitory activity. We performed a detailed analysis of the structural basis of ASIC1a inhibition by amiloride, using a combination of in silico approaches to visualize its interaction with the ion pore in the open state. An artificial activation (otherwise, expansion of the central pore) causes a complex modification of the channel structure, namely its transmembrane domain. The output protein conformations were used as a set of docking models, suitable for a high-throughput virtual screening of the Enamine chemical library. The outcome of the virtual screening was confirmed by electrophysiological assays with the best results shown for three hit compounds.

## 1. Introduction

Acid-sensing ion channels (ASICs) are proton-gated ion channels that are found throughout the mammalian nervous system. They belong to the epithelial sodium channel/degenerin (ENaC/DEG) family of amiloride-sensitive ion channels [1,2]. Although transmembrane ionic currents in response to extracellular acidification were first observed in 1980 [3], these channels received their modern name only after being cloned in 1997 [4]. Currently, at least ten subunits encoded by five different genes (ASIC1-5) are known. Splice variants of the ASIC1 gene give ASIC1a, ASIC1b, and ASIC1b2 subunits; ASIC2–ASIC2a and ASIC2b subunits [5,6,7,8]. ASIC3 encodes a single variant in rodents but three splice variants in humans (ASIC3a, ASIC3b, and ASIC3c) [9]. The splice variants for ASIC4–5 genes have not yet been described.

The ASIC subunits can form both homo- and heterotrimeric complexes, sharing a common protein structure characterized by two transmembrane domains, one extracellular loop, and intracellular N- and C-terminal domains. The large extracellular loop plays an essential role in the regulation and control of the ASIC channel activity, being a part of the large domain, which has some resemblance to a clenched hand, conditionally divided into palm, knuckle, finger, β-ball, and thumb [10]. Despite the information on the membrane topology being confirmed by the crystal structures of chicken ASIC1 (cASIC1), there is some difference in the sequence of the intracellular channel domain [11].

ASIC1a and ASIC3 homomers are found to be the most sensitive to extracellular protons, compared to other subunits and their combinations [6,11,12]. ASIC2b, ASIC4, and ASIC5 are not activated by protons at all [5,13]. It is more intriguing that, unlike ASIC2a, the heterologous expression of ASIC2b alone does not support proton-gated currents [14]. Within the nervous system, ASIC1a, ASIC2a, and ASIC2b [7,8,15] are primarily expressed in the CNS, while ASIC3, ASIC1b, and ASIC1b2 are in sensory neurons of the peripheral nervous system [16,17]. ASIC3 expression correlates well with its role in multimodal sensory perception [18,19], such as nociception, mechanosensation, and chemosensation. In contrast, an overlap in the expression of ASIC2 and ASIC1a proteins with synaptically dense regions in multiple brain structures is of special relevance, underscoring the importance of ASICs in synaptic function [20].

At the periphery, ASICs are typically expressed in sensory neurons that innervate tissues and organs and participate in sensory processes such as mechanotransduction, chemoreception, and nociception [21]. Brain ASICs have essential roles in the mechanisms of synaptic transmission, and synaptic plasticity (for a review, see [22]. The role of ASICs in higher-order activities such as learning and memory [2], and fear conditioning [23] is determined by their involvement in the functioning of synapses in the amygdala, hippocampus, striatum, and cingulate cortex [22]. ASICs also contribute to various diseases [24]. This is not surprising considering that tissue acidosis is a major contributor to neuronal cell death in many neurological diseases and ASICs, namely, subtype ASIC1a, are critical to acidosis-induced brain neuronal damage [25,26]. Pathologies involving ASICs include various pain states [27,28], ischemic stroke [25,29], epileptic seizures [27,30,31] degenerative disorders such as Alzheimer’s disease [32], multiple sclerosis [33,34], Parkinson’s disease [35], anxiety disorders [23] and even neonatal hyperbilirubinemia [36]. See [22,24,36] for detailed reviews.

Even though these channels are involved in various physiological processes in the central and peripheral nervous systems, as well as their intriguing features as a novel therapeutic target for diverse pathological disorders, the pharmacology of ASICs is currently underexplored. The first described molecule showing activity against ASICs was the weak and non-selective blocker amiloride [4,37]. The invention of natural peptides showing high affinity binding to the channels such as psalmotoxin 1 [38] and mambalgin 1 [39], together with the development of the crystal structure of the ASIC1a channel [10,40] allowed discovery of potent small molecule inhibitors: compound 5b [41], JNJ-799760 [42], and JNJ-67869386 [42]. These molecules bind to the acidic pocket of the channel mimicking the action of psalmotoxin 1. Unfortunately, the discovery of the ASIC3-specific peptide APETx2 [43] did not promote this channel’s invention of high-affinity modulators. There are several non-selective low-affinity modulators described to date: nafamostat [44], A-317567 [45], diminazene [46,47,48], other diarylamidines [49] and some non-amidine chemotypes [50,51]. Non-steroidal anti-inflammatory drugs (NSAIDs) are weak direct inhibitors of ASIC currents [52]. Several endogenous substances, for example, spermine [53], histamine, or corticosterone [54] can also modulate the activity of ASICs.

In this study, we combined both computational and electrophysiological approaches to investigate the regulation of these channels by 4-(Azolyl)-benzamidines derivatives, which were identified by virtual screening using Enamine’s stock chemical space. In the structure-based section that includes homology modeling, molecular dynamics (MD) simulation, and molecular design methods, we provide some results on different conformational and functional states, binding sites, and ligand interactions with the ion channel. The next stage assumed the implementation of electrophysiological techniques to confirm or refute activity predictions. A successful application of computer-aided modeling and drug design to identify potent compounds and further validate these pharmacological agents under physiological conditions appeared to be an effective strategy for optimal hit-to-lead development.

## 2. Results

Different literature and crystallographic data on ASIC suggested the presence of several functional binding sites, most of which were targeted to large molecules, in particular peptides and peptidomimetics [55,56,57]. The most notable of these represents the acidic pocket, a region of intra-subunit contacts that play a role in pH-dependent gating and binding of some modulators. From this standpoint, some inhibitors can prevent mutual motions of ASIC’s subunits or prohibit interaction with channel activity regulators (for example, single zinc and calcium ions), and other substances plugged the pore itself. Among all the deposited ASIC complexes the most important structures are amiloride-bound structure (4NTX) and complexes of ASIC1a with JNJ-799760 (6X9H) and Psalmotoxin (4FZ0) revealing molecular rearrangement details and distinct binding modes [42,58,59]. At the same time, amiloride and a group of diminazene derivatives are the most well-known small molecule effectors of the channel. The presence of a basic amidine or guanidine group seems to be critical for a high-potency inhibition of ASIC channels [50]. Unfortunately, there were no reports of other known and proven inhibitors in ChemblDb 27. Since the selectivity of the proposed reference inhibitors leaves a lot to wish for, as well as the ADME properties, we prepared screening libraries of Enamine ltd for the virtual search of promising hits. The overall database was filtered by physicochemical parameters.

**Figure 1:**
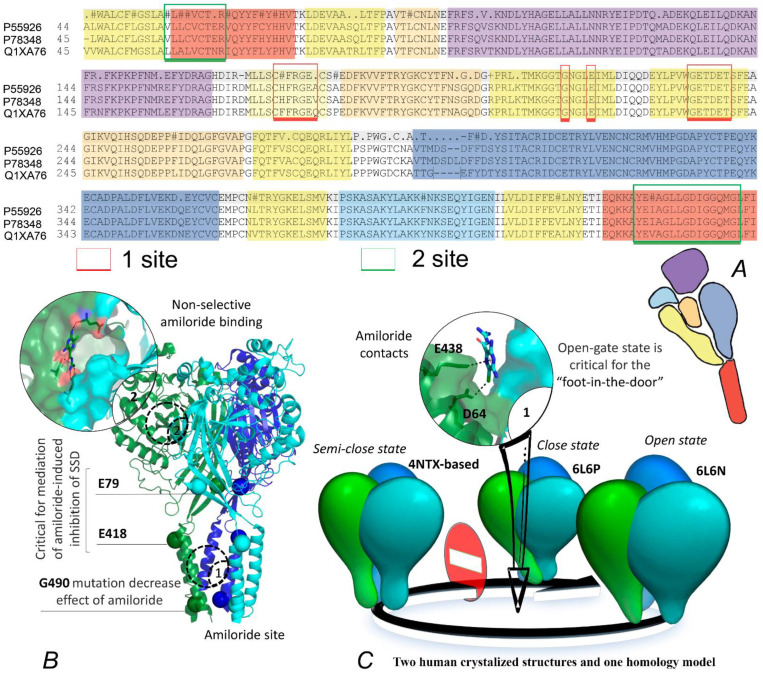
(**A**) Sequence alignment of Asic1a from *R. norvegicus* (P55926), *H. sapiens* (P783448) and *G. gallus* (Q1XA76) together with a schematic representation of the ASIC1 subunit divided into the different domains: finger (purple), knuckle (turquoise), β-ball (orange), palm (yellow), and thumb (blue). Two binding sites of amiloride are also mapped on the sequence alignment (signed with indices 1 and 2). (**B**) A ribbon representation of the human Asic1 homo-trimer (6L6N) with structural data, critical residues for the amiloride-associated effect detection, and its, already determined, binding sites and superposed ligands from 4NTX. (**C**) Mechanism of amiloride action against the open-state ASIC and its unusual interaction map inside the binding site.

The main dispute was centered on the exact place of the reference compounds on the surface, inducing a detectable inhibition effect (Figure 1). The controversy prevailed for a long time concerning the corresponding amiloride site, which could be located in the exodomain or the transmembrane site [58,60]. Apparently, it seems that binding sites on the surface of the extracellular domain are more suitable for long or volumetric molecular structures. In addition, previous computational studies on the interaction of dicationic compounds (nafamostat, DAPI, diminazene) with the ASIC1a structure suggested they are probably not pore blockers [49]. However, recent site-directed mutagenesis in Asic1 from R.norvegicus demonstrated, that residues E79 and E423 (E418 in humans) are critical for mediating the amiloride-induced inhibition of SSD, while the replacement of G445 (or G490 in human) impacts the inhibition effect of the drug [61].

Amiloride can hardly be called a site—or even target—specific ligand, showing its activity against different targets [62,63]. However, the results of crystallography and the above-described biochemical analysis, suggest that the transmembrane domain is a preferable target for the virtual screening (Figure 1). Importantly, the binding process must occur in the open state of the protein structure. It was proved with patch clamp studies, which indicated diminazene and amiloride primarily function as open-channel blockers [64]. That means that it would be more correct to investigate the interaction on different models by means of docking and MD. The pore-opening mechanism itself has already been proposed and accurately studied by means of molecular dynamics methods. Therefore, our task aimed to obtain the most appropriate site geometry for the docking procedure.

**Figure 2:**
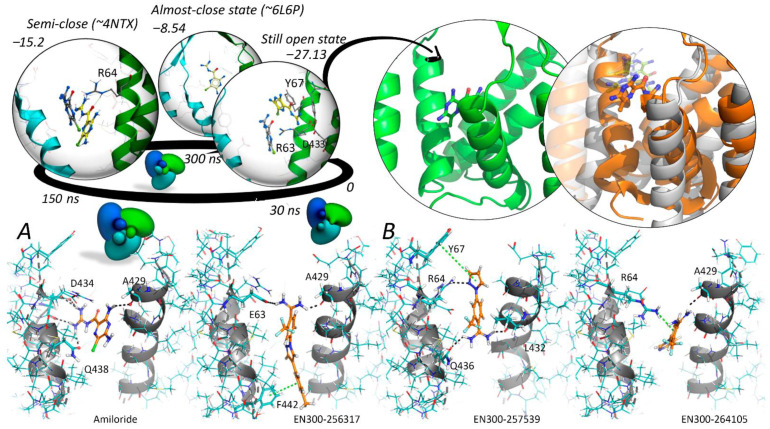
The most promising poses and high scores of the docked amiloride molecule in three important functional states, optimized by molecular dynamics. (**A**) The reference amiloride structure from 4NTX, aligned to the certain trajectory slice, is colored gray. The most adequate orientation and interactions of amiloride with the environment was shown for a slightly extended binding site from the semi-open state. The reference complex of 4NTX is colored green and the initial coordinate from the docking is grey colored. (**B**) The ligand finally took its place (orange color) not far from the source crystallized ligand.

We focused our attention on the transmembrane region and the interaction between adjusted secondary structures [65,66,67]. Our model objects were represented by three transmembrane domain states—built based on the open (6L6N) and closed (6L6P) human proteins and a semi-closed state of a chicken homologue protein (4NTX). The initial docking procedures in crystal structures revealed a discrepancy in the localization and orientation of the amiloride poses depending on the channel activity mode. In the closed and, interestingly, amiloride bound semi-closed state, the output poses didn’t match the reference co-crystallized ligand position. However, in the open state everything turned out better. At the same time, the delineated site is absent in the open state and is shaped with helices from adjacent chains. From there, we concluded that the amiloride binding could occur when the pore starts to constrict. To obtain a more extended binding site for each functional state we performed a targeted MD simulation. After the assignment of two endpoints (6L6N and 6L6P) we performed two short relaxation simulations. Both relaxed structures were then processed by the RMSD-based protocol of enforced simulation. Starting from the open state, we gradually brought the trimer to the completely closed one, having passed all the stages of transmembrane helix kinks. The trajectory of transmembrane domain motions was clustered using a k-means algorithm with a predetermined number (15) of clusters. These clusters covered the entire conformational space and when we superposed the centroid structures of each cluster to the 4NTX crystal structure, we found an almost similar conformation after the middle of the track (nine clusters). The simulation provided some information about the moderate binding site volume changes and side-chain motion. Then we started an Induced Fit Docking in these 15 centroid structures, which revealed that the most appropriate amiloride binding mode corresponds to the period, when the pore ‘blades’ are passing a so-called semi-closed state (7). The compound interacts with a lipid environment and forms a Pi-cation stacking interaction and salt bridge (Figure 2A).

Of course, that gives the ligand more degrees of freedom during the conformation search (Figure 2B). The molecule of amiloride was surrounded by residues D434, G436, G437, Q438, Q436, G440, A429 and L441. Hydrogen bonds were formed between amiloride and the backbone of A429 and with the side-chains of D434 and Q438. Hydrophobic contacts were observed with residues G437, R64, G440, and L441.

We used the conformation to launch a virtual screening of the pre-filtered Enamine database, yielding a group of substances that satisfied the previously described pharmacophore model. The contact map was formed with at least two residues from the closest environment (D434, R63, and E62), involved in the donor/acceptor interactions, while the most critical stacking interaction was provided with the tighter fit of the Y67 ring plane and, in some cases, Y425 ring (Figure 2B). These docked compounds were specifically oriented inside the binding site, positioning the most hydrophobic groups close to the surrounding lipid molecules.

**Figure 3:**
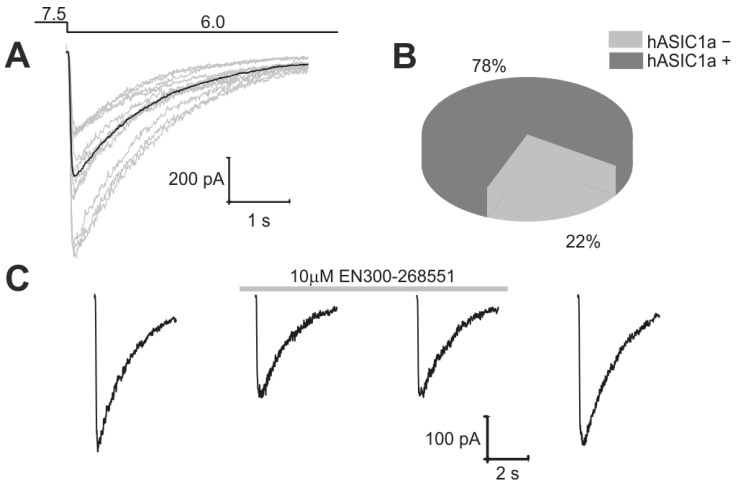
Manual patch-clamp screening procedure. (**A**) Examples of hASIC1a current traces in HEK 293 cells elicited by a pH drop from 7.5 to 6.0 at holding potential −70 mV (black line represents the averaged current trace). (**B**) Summary data for the responsiveness of HEK 293 cells to a pH drop. (**C**) Standard experiment showing the evaluation of the inhibition potency of the substance.

Next, we evaluated the selected compounds against low pH-induced currents in native human ASIC1a channels, naturally expressed in HEK 293 cells [41,68]. In our experimental conditions, approximately 80% of cells displayed ionic currents through hASIC1a channels, primarily mediated by sodium ions, in response to a rapid pH drop from 7.5 to 6.0 (Figure 3A,B). The amplitudes of these currents ranged from 200 to 1500 pA when measured at a holding potential of −70 mV. With 3 min intervals between applications of acidic solutions, these currents remained stable for at least 30 min, providing a sufficient timeframe to assess the activity of a chosen substance. All tested compounds failed to elicit any discernible transmembrane current upon application to the cell; instead, they induced a significant but reversible inhibition of acid-induced current (refer to Figure 3C and Table 1).

As a result of the initial screening procedure, we found that all tested compounds at 100 µM concentration significantly and reversibly inhibited the hASIC1a currents induced by a rapid pH drop from 7.5 to 6.0 (see Table 1).

**Figure 4:**
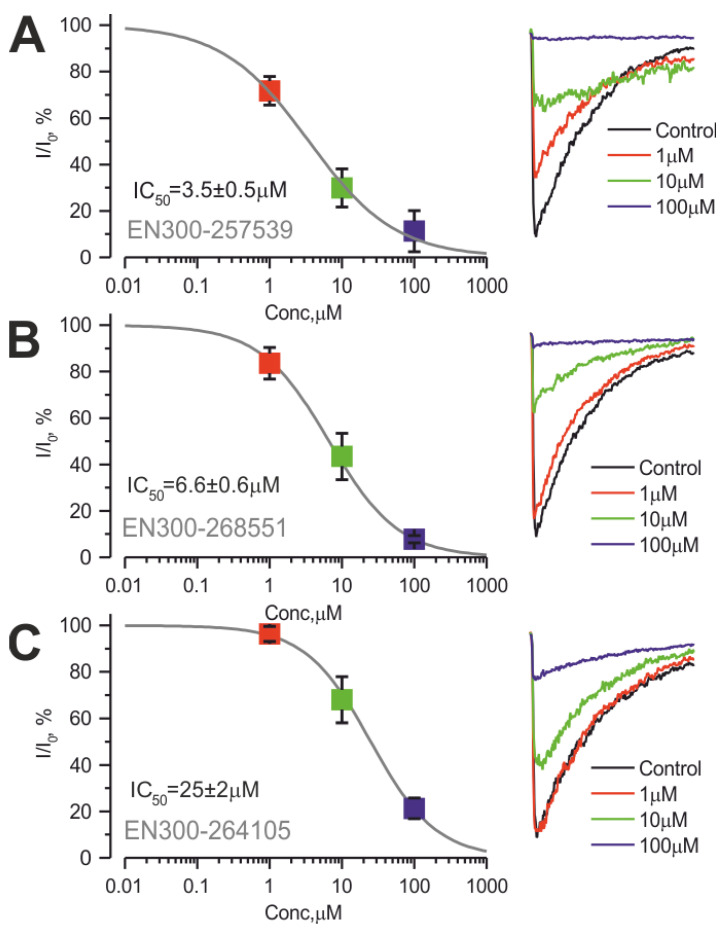
The most effective inhibitors of hASIC1a currents. (**A**) left panel: dose–effect curve for EN300-257539 compound (estimated IC_50_ was 3.5 ± 0.5 µM); right panel: representative normalized currents traces for indicated concentrations. (**B**) left panel: dose–effect curve for EN300-268551 compound; right panel: representative normalized currents traces for indicated concentrations. (**C**) left panel: dose–effect curve for EN300-264105 compound; right panel: representative normalized currents traces for indicated concentrations. n = 4–5 cells.

The most potent ones, EN300-257539, EN300-264105, and EN300-268551, exhibited near-complete inhibition of ASIC1a currents at this concentration, with corresponding half-maximal inhibition concentrations (IC_50_) estimated as 3.5 ± 0.5 µM, 6.0 ± 0.6 µM, and 25 ± 2 µM, respectively (see Figure 4 for details). Such compound activity is comparable with well-known ASIC1 antagonists such as amiloride [4], nafamostat [44], benzamil [4], ethylisopropylamiloride [4] and diminazene [47].

Since EN300-257539 emerged as the most potent compound in this series, it was selected for further investigations. Subsequently, we assessed whether these compounds, which were predicted to bind to the pocket in the transmembrane domain in silico, exhibit selectivity among different ASIC subunit subtypes. To manage costs and time these experiments were conducted on native ASIC currents through channels expressed in various types of rat neurons, including hippocampal, cortical, and DRG neurons.

**Figure 5:**
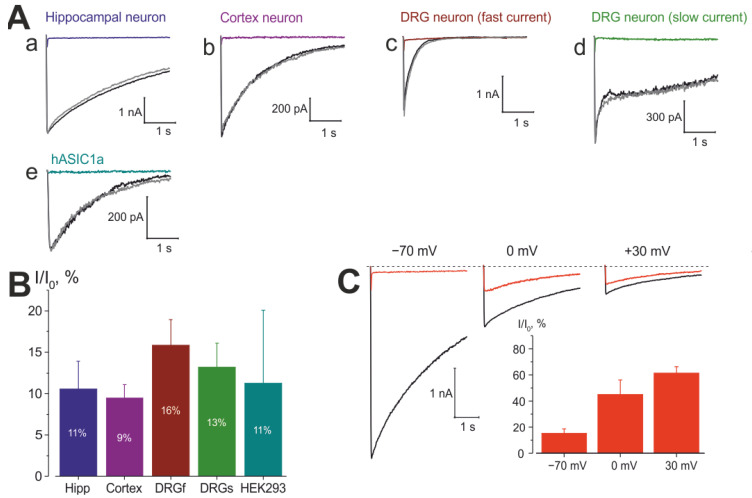
The inhibition of ASICs by EN300-257539 is subtype-insensitive but depends on cell membrane voltage. (**A**) ASIC current traces through the channels expressed in various neurons clamped at −70 mV. In all cases currents were elicited by a rapid pH from 7.5 to 6.0; (**a**) representative traces for hippocampal neurons: black trace represents control response, grey—recovery after washing out of the substance, colored—result of substance action; (**b**) the same as for (**a**), but for cortical neurons; (**c**) the same as for (**a**), but for fast current in DRG neurons; (**d**) the same as for (**a**), but for slow current in DRG neurons; (**e**) the same as for (**a**), but for hASIC1a current in HEK 293 cells. (**B**) Summary for the data presented in (**A**). (**C**) Current traces at indicated holding voltages, black control ones, red—under the substance. Inset shows the summary for inhibition at different membrane voltages.

In rats, these cells predominantly express rASIC1a in the hippocampus; rASIC1a, rASIC2a, rASIC2b in the cortex; and rASIC3, rASIC1a, rASIC1b [69]. These subunits can form functional homomeric and heteromeric channels with distinct pharmacological properties. Our investigation revealed that the inhibition of ASIC currents by 100 µM EN300-257539 was consistently similar (refer to Figure 5Aa–d,B) across different rat tissues (10.55 ± 3.37% (n = 5) for hippocampal neurons, 9.45 ± 1.66% (n = 6) for cortical neurons, 15.87 ± 3.09% (n = 4) for fast ASIC currents in DRG neurons, 13.21 ± 2.86% (n = 5) for slow currents in DRGs neurons) compared to the inhibition of hASIC1a current (11.27 ± 8.80% (n = 5) in HEK 293 cells (Figure 5Ae,B)). This suggests that this molecule may not exhibit specificity across different ASIC subtypes and channels from different species.

The suggested binding pocket for the described molecules is located in the transmembrane region of the channel. Therefore, their inhibition should exhibit dependence on membrane voltage, similar to what has been described for amiloride on ASICs [37] and ENaCs [70]. Indeed, the observed inhibition of ASIC currents by EN300-257539 demonstrated voltage-dependent properties: at −70 mV 85 ± 3% (n = 4) of a whole current was inhibited by the molecule, increasing voltage to 0 mV resulted only in 55 ± 11% (n = 4, p = 0.01, two-tailed two-sample unequal variance Student’s *t*-test) of inhibited fraction of the current, further increase the voltage to +30 mV led to a decrease in this fraction to 39 ± 5% (n = 4, p = 0.05, two-tailed two-sample unequal variance Student’s *t*-test). These data indicate that the binding pocket of the molecule is located in the membrane region of the ASIC channel.

It should be noted that the most active substances (EN300-257539, EN300-264105, and EN300-268551) contain a CF3 group, involved in interactions with the cell membrane. While the docking output presented a few alternative poses for these compounds that did not make contact with the membrane, they exhibited lower docking scoring values. We observed that the presence of the functional group caused some bending in the overall structure, making it more conducive to the formation of stacking contacts. Our experimental data suggest that the absence of this group hurt inhibitory activity.

## 3. Discussion

Numerous studies suggest that amiloride and the group of diminazene derivatives represent one of the few low-molecular-weight effectors of the ASIC channel. In addition, all the compounds are not optimal by the availability profile. However, for now, there are no available analogs in the chemical databases. Recently some novel analogs were suggested, using the isometric replacement of the amidine group, which usually causes a decrease in activity [71,72,73,74]. As opposed to this, synthesis and validation are also demanding. In general, such comprehensive projects, combining several approaches with specific techniques, are very sensitive to incoming data from the previous stage. From these, the most critical step to identify a proper protein conformation required at the interaction of the inhibitor in the binding pocket appears crucial. An interesting example, confirming the statement, is a previous study on the identification of the ASIC1 effectors. Initially, the ligands were docked into another ASIC1 binding site. Still, after obtaining the biological data, the search area was changed, which allowed us to suggest the interpretation for this mechanism of interaction [41].

Thus, the main efforts were initially targeted at identifying the functional binding sites in the corresponding protein conformations and their subsequent interaction with the inhibitor. Curiously, the molecular docking technique revealed a low-affinity rate of the amiloride inhibitor to the protein in the crystallized open and closed states. This disappointing situation challenged us to extend a standard protocol of in silico workflow. Taking into account the fact that the protein should be activated to bind amiloride, we performed several rounds of molecular dynamics and molecular docking analysis to obtain the initial structure of the protein–ligand complex, which we employed to construct a full-fledged docking model. Consequently, a set of residues that form hydrogen bonds and engage in stacking interactions was identified. These data helped in conducting and analyzing the results of the virtual high-throughput screening.

Further computational procedures resulted in a set of compounds with a predicted possibility of protein binding, causing its subsequent dysfunction. These compounds demonstrated similar interaction with residues of the transmembrane domain, as was shown for amiloride. Finally, a fluorinated CF_3_ group, found in a number of the high-scoring compounds was evidently shown to increase the probability of binding in such a specific pocket, due to its effect on the structure and additional interaction with the surrounding membrane. In the physiological tests, the absence of the CF_3_ group in the ligand structure clearly affected the inhibitory activity negatively.

All in all, this project offered us new scaffolds and the possibility of introducing chemical modifications to obtain various combinations of features. Beyond that, a validated workable computational model of the process will allow us to predict the probability of binding the proposed inhibitors to the binding site quickly and reasonably well. But despite achieving some rapid results, all of this provides plans to optimize the process, particularly in preparing a sample of substances for experimental work, which is the most sensitive and time-consuming. In the meantime, the results of this project represent a launch step for a future, larger-scale MedChem project.

## 4. Materials and Methods

### 4.1. ASIC1 Model

ASIC1 sequences from chicken and human homologues share 90% of identity, based on the sequence alignments in ClustalW 2.0.12 (Conway Institute UCD Dublin, Ireland) [75]. Thus, we used human closed (PDB: 6L6P) and open (PDB: 6L6N) forms to execute the modeling while a homology model of built from the semi-closed form from *Gallus gallus* (4NTX) became an additional structural milestone [58].

### 4.2. MD Simulation

Based on the represented protein structures we rebuilt three corresponding functional modes of the complete human protein structure by homology modeling. For the relaxation step we used data on the protonation, calculated in a recent study [76]. The following residues, where pKa was close to 5.3 were protonated: E63, H70, H72, E97, H110, E113, H173, E177, E219, E238, E254, E321, D347, D351, E355, D409, E413, E418, D434, E452. Then we applied a Charmm-gui server (24/03/2020) [77] and then both models were embedded in a phospholipid bilayer. We composed two systems formed with mono lipid membrane containing 374 molecules of POPC (1-palmitoyl-2-oleoyl-sn-glycero-3-phosphocholine) and TIP3P water model clusters [78]. All-atom MD simulations were performed with the CHARMM36 force field [79] and Gromacs 2020 (Science for Life Laboratorym, Stockholm, Sweden) [49]. After energy minimization and equilibration, the system was simulated for 50 ns using Gromacs 2020 [80] and a timestep of 2 fs. Long-range electrostatic interactions were calculated using the particle mesh Ewald method and hydrogen-bond length was constrained using LINCS [81]. Pressure and temperature values were maintained through the use of the Parrinello–Rahman [82] barostat and v-rescale [83] thermostat, respectively.

Targeted MD simulation of 300 ns was performed using the GENESIS MD program [84]. Constraints were assigned to all bonds involving hydrogen atoms and water molecules were kept rigid. Long-range electrostatic interactions were calculated using the particle-mesh Ewald summation method. Lennar–Jones interactions were smoothed over a 10-12 Å range with a force-based switching function. Multiple time-step integration was used for calculating long-range electrostatic interactions. The time-step of the MD integration was 2.5 fs. Long-range interactions were evaluated every two steps. Temperature (310.15 K) and pressure (1 atm) were controlled by the stochastic rescaling thermostat combined with the MTK barostat proposed by Bussi et al. [83].

Three full-atom systems became starting points for separate simulation runs. Each system’s trajectory length of 100 ns was sufficient to obtain a relaxed conformation (mutual helix disposition and membrane-mediated constraining). The level of pore clearance and the overall geometry were tested with a multifunctional tool Caver Analyst 2.0 (Masaryk University. Czech Republic), which is incredibly convenient for the identification of internal implied movement [85,86,87,88,89].

We focused on the channel residues and the second step of the simulation was studied using a k-means clustering algorithm with a predetermined number of clusters. These 15 clusters covered the entire conformational space almost in equal shares. Then the centroid molecules of each cluster were aligned to the crystal structures, providing some information about the moderate binding site volume changes and side-chain motions.

### 4.3. Molecular Docking and a High-Throughput Virtual Screening

The initial receptor grids for a standard flexible docking run were specified from the residues, involved in ligand binding (based on the 4NTX structure) transferred then to the structures, corresponding to already optimized open, closed, and semi-open states [90]. But neither of the models showed high scores, even a 4NTX-based homology model. Then an induced Fit Docking procedure was conducted to put amiloride in each of three protein functional modes, after preprocessing via ICM Molsoft. Amiloride was structurally preprocessed directly in the docking process, while the Enamine screening library was previously indexed. The scaling factors of the accuracy for the virtual screening were altered from the initial docking value of 5.0 to 3.0. The Enamine screening library was pre-filtered and indexed and passed through the docking model. A similar protocol was applied recently in the same project [91].

### 4.4. HEK 293 Cell Culture

Human embryonic kidney 293 (HEK 293) cells (American Type Culture Collection, Manassas, VA, USA) expressing endogenous hASIC1a channels [68] were cultured in Dulbecco’s modified Eagle medium (DMEM) supplemented with 10% fetal bovine serum (FBS) and 10 U/mL penicillin and 10 mg/mL streptomycin. Dissociated cells were either re-plated for a new passage or used for patch clamp experiments. Cells were cultured by standard procedure [92] at 37 °C under an atmosphere of 5% CO_2_ and 95% air with approximately 95% humidity.

### 4.5. Animals

Wistar rats aged 8–14 days postnatal were used throughout the study. Animals were obtained from the animal facility of Bogomoletz Institute of Physiology (Kyiv, Ukraine), housed on a 12 h light–dark schedule, and given food and water ad libitum. The following experimental procedures were performed by the Institute’s Bioethics Committee guidelines.

### 4.6. Acute Isolated Rat Hippocampal and Cortical Neurons

Combined entorhinal cortex/hippocampal slices including neocortical areas (Te2 and Te3), entorhinal cortex, subiculum, and hippocampus were prepared from Wistar rats aged 12 to 14 days postnatally (P12–14) as previously described with some modifications [93]. On the day of the experiment, the rat was deeply anesthetized using isoflurane and subsequently decapitated. The brain was removed and placed into ice-cold oxygenated (95% O_2_-5% CO_2_) artificial cerebrospinal fluid (ACSF) of the following composition (mM): NaCl 119, KCl 2.5, CaCl_2_ 2.0, MgSO_4_ 1.3, NaHCO_3_ 26, NaH_2_PO_4_ 1.2, and glucose 11 (pH 7.35). Cerebellum, frontal lobe region (coronal section), and ventral–lateral areas (sections at an angle of 20° to 30° off the horizontal axis) were removed from the brain. The remaining part of the brain was mounted on the stage of a Vibroslice NVSL (World Precision Instruments Inc., Sarasota, FL, USA) and cut (300 μm) through the hemispheres at an angle of 30–35° of their horizontal planes. Slices were preincubated in this solution for 30 min at room temperature. Enzymatic treatment proceeded in the same solution with a lower Ca^2+^ concentration (0.5 mM) and containing 0.5% protease from *Aspergillus oryzae*. Incubation in the enzyme solution proceeded at 32 °C for 30–40 min. Slices were subsequently kept in an enzyme-free solution containing normal Ca^2+^ concentration and used within 6–8 h to obtain isolated neurons.

For the isolation procedure the slice was transferred to an extracellular solution containing (mM): NaCl, 150; KCl, 5; CaCl_2_, 2; MgCl_2_, 2; HEPES, 10; pH adjusted with NaOH to 7.4. The cortex area was separated from slices under microscope control with sharp steel needles. Cells were isolated by successive trituration of small pieces of the slice through several fire-polished pipettes, with the opening diameter decreasing from 500 to 100 μm [94]. The rest of the slice was used for hippocampal cell dissociation. Individual cells were mechanically isolated from the CA1 region of the hippocampal slices by the vibrodissociation method [95]. Acute-isolated neurons were identified by their characteristic shape, they had diameters of 10–30 μm and preserved small dendritic arborization. After isolation, they were usually suitable for recording for 2–4 h.

### 4.7. Primary Culture of Rat DRG Neurons

Primary culture of dorsal root ganglia (DRG) neurons was prepared from 8–12-day-old Wistar rats of both sexes [92]. After decapitation, ganglia were rapidly removed and transferred into minimum essential medium Eagle’s solution (MEM) containing 4 mg/mL trypsin and 2 mg/mL collagenase, where they were incubated for 25 min at 35 °C. Throughout the entire procedure, the medium was continuously saturated with a 95% O_2_ and 5% CO_2_ gas mixture to maintain pH 7.4. After the enzyme treatment, ganglia were washed out and dissociated in the MEM solution containing 10 mM of HEPES–NaOH, pH 7.4. Suspension of isolated neurons was transferred into 25 mm Petri dishes containing 90% Dulbecco’s modified Eagle’s medium (DMEM), 0.3% penicillin, 10 μg/mL insulin, and 10% heat-inactivated fetal calf serum (pH 7.4; when incubated in 95% air/5% CO_2_). After maintaining for 5–48 h at 37 °C, the cells were used in electrophysiological experiments.

### 4.8. Electrophysiological Recordings

Currents through ASIC channels were elicited by a rapid drop in extracellular pH from 7.5 to 6.0 and recorded at a holding potential of −70 mV, unless otherwise indicated. The solution exchange velocity was set to approximately 20 ms (measured by exchanging extracellular buffer with a partial salt solution containing 50% extracellular buffer and 50% H_2_O, open-tip protocol, see [96] for ref. Whole-cell patch clamp recordings were made with the EPC-8/LIH 1600 amplifier/acquisition system. The current traces were sampled at 10 kHz and filtered online at 3 kHz. Experimental data were collected using PatchMaster v2.53 software (all from HEKA, Lambrecht/Pfalz, Germany). Patch electrodes (2–3 MOhm) were filled with a solution containing (in mM): 120 KF, 20 Tris-Cl (adjusted to pH 7.3 with KOH). The extracellular solution contained (in mM): 130 NaCl, 5 KCl, 2 MgCl_2_, 2 CaCl_2_, 20 HEPES/NaOH, pH 7.4. A fully automated “jumping table” set-up (PharmaRobot, Kyiv, Ukraine) was used to change extracellular solutions (see [97] for reference).

### 4.9. Data Analysis

Offline analysis was conducted using Prism 5 software (GraphPad, La Jolla, CA, USA). The peak amplitudes of a current were obtained by subtracting the baseline current from the peak current values. Each concentration data point was obtained by averaging ratios of peak current amplitudes to their corresponding current amplitudes in the control conditions across different cells. As a result of such normalization, 100% corresponds to control conditions with no drug applied to the cell, whereas 0% reflects the total current inhibition caused by the substance. The results were expressed as mean ± standard deviation (SD); ‘n’ denotes the number of recordings. Due to the described above normalization procedure, the 50% inhibitory concentrations (IC_50_s) can be estimated through nonlinear regression analysis employing the simplified Hill-Slope equation: $y=\frac{100}{{1+(\frac{x}{{IC}_{50}})}^{p}}$, where ‘*p*’ denotes the Hill coefficient.

### 4.10. Drugs and Chemicals

All compounds were obtained from the Enamine Ltd. (Kyiv, Ukraine) store. Unless otherwise noted, all chemicals were purchased from Sigma-Aldrich Chemie GmbH (Taufkirchen, Germany). DMEM, FBS, penicillin, and streptomycin solutions were purchased from Thermo Fisher Scientific (Waltham, MA, USA). All compounds were dissolved in DMSO and stored at 4 °C as stock solutions before being dissolved to the desired concentration in the extracellular solution just before the experiment.

## 5. Conclusions

**Figure 6:**
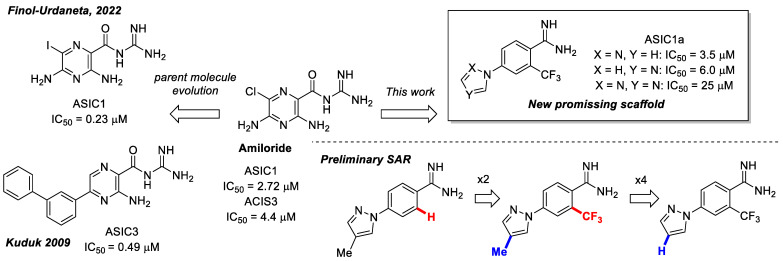
Finalized outcome of the study represented in the form of a chemical flow charts: initial ancestor and its evolution based on selected papers and our findings [51,74].

This study has several important outcomes. First, a detailed analysis of the inhibition of ASIC1a by amiloride, demonstrating how it interacts with the open state of ASIC1a within the ion pore above the “GAS belt”. Second, we applied a complex approach to induce collective motions of the TM domain substructures and investigated the reasons for its functional disorder in response to the ligand binding. Building on these efforts we formed a novel interaction map and then developed a relevant virtual screening model. After this fundamental step, we performed a high-throughput virtual screening of the Enamine screening library. In contrast to the well-published efforts of Merck [49] and recently published Kuduk and Finol–Urdaneta works [51, 74] directed to the evolution of the parent amiloride molecule, the current in silico investigation discloses a new scaffold (Figure 6). The quality of our in silico model was further confirmed by electrophysiological assay resulting in the identification of three hit compounds. Especially important, we built a preliminary structure–activity relationship from the in vitro investigation of eight compounds and determined the principal features, responsible for their activities. Firstly, appearance of the lipophilic CF_3_-group in the *ortho*-position towards amidine in the benzene ring is critical. Secondly, unexpectedly the “reverse” magic methyl effect [98] was observed in the azole ring. Any small substituent, including a methyl group that is installed on the ring causes the activity to be decreased. We took into account the relatively “small size” of the 4-(azolyl)-benzamidine scaffold, the latest attractive chemotype for the subsequent drug discovery.

## Figures and Tables

**Table 1:** The activity of chosen substances.

Substance	Structure	Score	I/Io (Mean ± SD), %	Recovery (Mean ± SD), %
EN300-257412	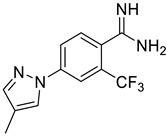	−19.46	37 ± 8	101 ± 7
**EN300-257539**	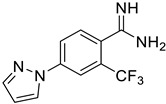	**−** **17.05**	**11 ± 9**	**101 ± 2**
EN300-256317	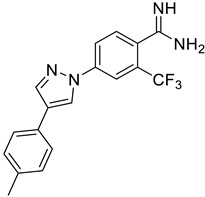	−19.53	7 ± 2	55 ± 2
EN300-262584	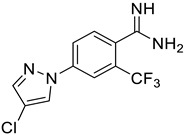	−17.18	19 ± 8	92 ± 4
**EN300-264105**	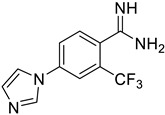	**−** **21.45**	**8 ± 2**	**97 ± 8**
EN300-256318	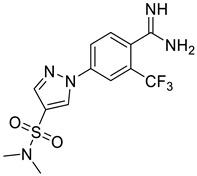	−18.59	76 ± 7	82 ± 5
EN300-2324118	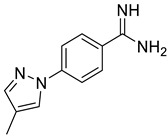	−19.46	65 ± 3	83 ± 15
**EN300-268551**	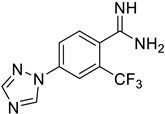	**−** **17.58**	**21 ± 4**	**102 ± 11**

## Data Availability

Data are contained within the article.
